# Biosafety studies of carrier cells infected with a replication-competent adenovirus introduced by *IAI.3B* promoter

**DOI:** 10.1038/mtm.2014.19

**Published:** 2014-05-28

**Authors:** Katsuyuki Hamada, Toshiro Shirakawa, Shuji Terao, Akinobu Gotoh, Kenzaburo Tani, Wenlin Huang

**Affiliations:** 1 Department of Obstetrics and Gynecology, School of Medicine, Ehime University, Shitsukawa, Toon, Ehime Japan; 2 Division of Infectious Disease Control, International Center for Medical Research and Treatment, Faculty of Medicine, Kobe University Graduate School of Medicine, Kusunoki, Chuo-ku, Kobe, Hyogo, Japan; 3 Laboratory of Cell and Gene Therapy, Institute of Advanced Medical Sciences, Hyogo College of Medicine, Mukogawa, Nishinomiya, Hyogo, Japan; 4 Department of Advanced Molecular and Cell Therapy, Kyushu University Hospital, Kyushu University, Maidashi, Higashi-ku, Fukuoka, Japan; 5 Department of Molecular Virology, Cancer Center, Sun Yat-sen University, Guangdong, China

## Abstract

The use of carrier cells infected with oncolytic viruses in cancer gene therapy is an attractive method because it can overcome viral immunogenicity and induce tumor immunity and significant antitumor activity. To enable human clinical trials of this treatment, acute and chronic toxicity tests must first be performed to ensure safety. *IAI.3B* promoter, oncolytic adenovirus AdE3-*IAI.3B* introduced by *IAI.3B* promoter, and A549 carrier cells infected with AdE3-*IAI.3B* were highly active in cancer cells but not in normal cells. Freeze-thawing increased the antitumor effect of A549 carrier cells by promoting the translocation of oncolytic adenovirus particles from the nucleus to the cytoplasm following the rupture of the nuclear membranes. No deaths or abnormal blood test data resulted from acute toxicity tests conducted in nude mice after a single dose. In chronic toxicity tests in rabbits, there were no serious side effects after eight doses of 1.25 × 10^7^ cells/kg or less for 4 weeks; a significant immune response is known to elicit increased numbers of antiadenovirus antibodies and enlarge the spleen. From these results, it could be concluded that cancer gene therapy of recurrent solid tumors using carrier cells can be safely trialed in humans.

## Introduction

More than 800 clinical trials of cancer gene therapies have been conducted to date, but encouraging clinical results have yet to be obtained. Recently, replication-competent viral vectors have been developed to improve antitumor activity. However, there remain two major concerns with the use of these viral vectors: frequent relapse of tumors despite temporal inhibition of tumor progression^1^ and generation of high titers of neutralizing antibodies that subsequently inhibit repetitive viral infection.^2^ Repetitive infection is difficult to achieve, although anti-CD3 antibody,^2^ polyethylene glycol,^3^ liposome,^4^ cyclophosphamide,^5^ and etoposide^6^ have been reported to overcome the humoral immune responses to viral vectors.

Many studies of replication-competent virus-infected carrier cells have been described: these include PA-1 ovarian cancer cells infected with oncolytic HSV-1,^7^ mesenchymal stem cells infected with oncolytic adenovirus,^8^ myeloma cells infected with oncolytic measles virus, vaccinia virus, vesicular stomatitis virus, coxsackievirus A21,^9^ cytokine-induced killer cells infected with modified vaccinia virus,^10^ rat hepatoma cells infected with oncolytic parvovirus,^11^ and autologous CD8^+^ lymphocytes infected with oncolytic vesicular stomatitis virus.^12^ However, the antitumor effect of these carrier cells was not of sufficient potency to kill cancer cells completely, since these carrier cells could not produce high enough virus titers and were vulnerable to damage even before they could kill the target cancer cells. A549 cells have been used conventionally in the production of various viruses containing adenovirus because of their high virus production capacity; thus, A549 carrier cells infected with oncolytic adenovirus show a significant antitumor effect in immunocompromised mice.^13^ A549 carrier cells also show the significant antitumor effect in immunocompetent mice, because they can overcome the infection inhibition of oncolytic adenovirus by the antiadenovirus antibody production.^13^

To enable clinical trials of type 5 adenovirus vectors to be undertaken, toxicity tests were reported in mice,^14^ dogs,^15^ and monkeys^16^ for nonreplicative vectors, and in mice^17^ and cats^18^ for replicative vectors to establish the safety of these vectors. The safety and efficacy of autologous and allogeneic cell-based adenoviral vector GVAX vaccines have been reported in non–small-cell lung cancer,^19^ but toxicity studies in experimental animals have not yet been reported. In addition, the biodistribution of oncolytic adenovirus-infected neural stem cells has been reported in glioma,^20^ but there are no reports of toxicity tests on carrier cells infected with oncolytic viruses including oncolytic adenovirus.

The *IAI.3B* gene was originally isolated from a high–molecular-weight fraction derived from ovarian cancer.^21^ Its promoter activity is very high in ovarian cancer, and a replication-competent oncolytic adenovirus named AdE3*-IAI.3B*, in which the *E1A* gene is under the control of the human *IAI.3B* promoter, replicates as efficiently as the wild-type adenovirus in ovarian cancer cells but not in normal cells.^22^ Although AdE3*-IAI.3B* temporarily induces the complete reduction of ovarian cancer cell tumors, ovarian tumors regrew because of its insufficient antitumor effects.^22^ In a previous study,^13^ human non–small-cell lung cancer A549 carrier cells were infected with AdE3*-IAI.3B*, and adenoviral particle–containing cell fragments derived from the carrier cells were engulfed by target cancer cells. This novel non–receptor-mediated adenoviral transfection system circumvents neutralization by antiadenovirus antibodies and enhances antitumor activity by the induction of antiadenoviral cytotoxic T lymphocyte responses after preimmunization with adenovirus in immunocompetent mice, and then, antitumoral immune response was induced. In this study, we examined the activities of *IAI.3B* promoter, AdE3-*IAI.3B*– and AdE3-*IAI.3B–*infected carrier cells in ovarian cancer cells, and other cancer cells. We performed acute and chronic toxicity tests of AdE3-*IAI.3B*–infected carrier cells in mice and rabbits, respectively, and investigated the infection and storage conditions appropriate for carrier cells as cellular biological drugs to enable clinical trials to be commenced.

## Results

### *IAI.3B* promoter, oncolytic adenovirus AdE3-*IAI.3B*, and A549 carrier cells infected with AdE3-*IAI.3B* are activated in cancer cells

**Figure 1:**
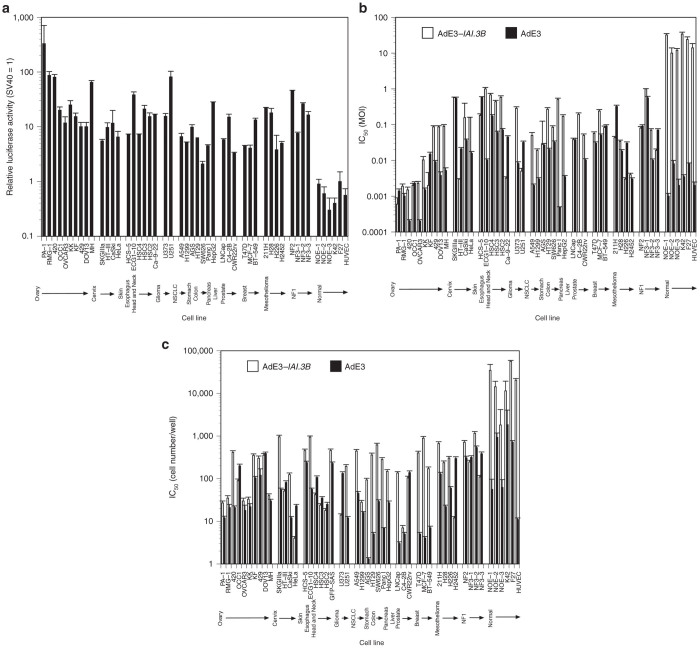
Tissue specificity of *IAI.3B* promoter, AdE3-*IAI.3B* and AdE3-*IAI.3B*-infected A549 carrier cells. (**a**) Transcriptional activity of the *IAI.3B* promoter in ovarian cancer, other cancer and normal cell lines. Bars, +SDs. (**b**) Cytotoxicity of AdE3-*IAI.3B* and AdE3 in ovarian cancer, other cancer and normal cell lines. Bars, +SDs. IC50, 50% inhibition rate of cell growth. (**c**) Cytotoxicity of A549 carrier cells infected with AdE3-*IAI.3B* and AdE3 in ovarian cancer, other cancer and normal cell lines. Bars, +SDs. IC_50_, 50% inhibition rate of cell growth.

To compare the transcriptional activity of the *IAI.3B* promoter in cancer and normal cells, its promoter activity was estimated by taking *SV40* promoter activity to be 1 (Figure 1a). *IAI.3B* promoter activity in all cancer cells was 40 times greater than that in normal cells, and that in ovarian cancer cells was 5 and 104 times greater than that in other cancer (*P <* 0.05) and normal cells (*P <* 0.01), respectively.

The antitumor activity of AdE3-*IAI.3B* in cancer and normal cells was investigated by determining the 50% inhibition rate of cell growth. Wild-type adenovirus AdE3 killed cancer and normal cells, while AdE3-*IAI.3B* killed all cancer cells as efficiently as AdE3 but not normal cells. AdE3-*IAI.3B* killed ovarian cancer cells 7.6 and 740 times more efficiently compared with other cancer (*P <* 0.05) and normal cells (*P* < 0.001), respectively (Figure 1b).

To compare the antitumor effect of AdE3-*IAI.3B-*infected A549 carrier cells in cancer and normal cells, we determined the 50% inhibition rate of cell growth. AdE3-infected A549 cells killed cancer and normal cells, but AdE3-*IAI.3B*–infected A549 carrier cells selectively killed cancer cells but not normal cells (*P <* 0.05; Figure 1c).

### Determination of infection and storage conditions for A549 carrier cells

**Figure 2:**
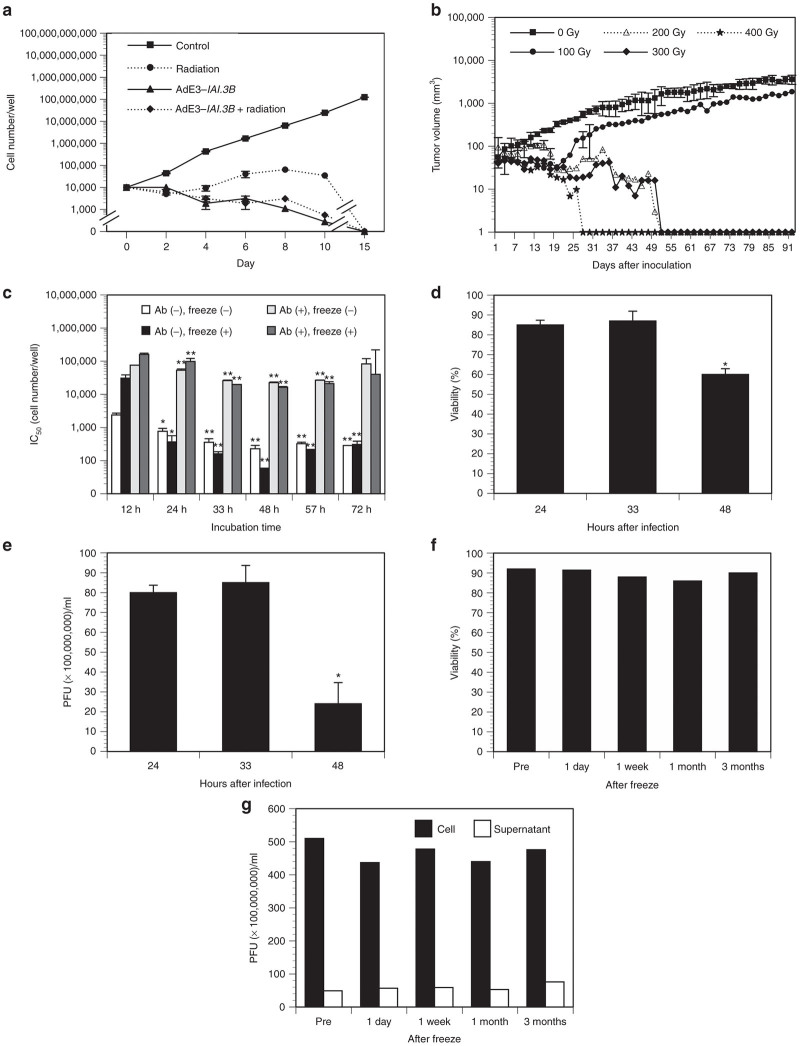
Infection and storage condition of AdE3-*IAI.3B*-infected A549 carrier cells. (**a**) *In vitro* growth inhibitory effect of A549 carrier cells infected with AdE3-*IAI.3B* at 200 MOI and irradiated at 200 Gy. (**b**) Effects of radiation exposure on tumorigenicity of A549 cells subcutaneously transplanted into nude mice (*n* = 5 per group). (**c**) Effects of infection time, antiadenovirus antibodies and freeze-thawing on the cytotoxicity of AdE3-*IAI.3B*-infected A549 carrier cells in ovarian cancer HEY cells. *P < 0.05; **P < 0.01. (**d**) Changes in viability of AdE3-*IAI.3B*-infected A549 carrier cells by infection time. *P < 0.05. (**e**) Changes of PFU activity of AdE3-*IAI.3B*-infected A549 carrier cells by infection time. *P < 0.05. (**f**) Changes of viability of AdE3-*IAI.3B*-infected A549 carrier cells following liquid nitrogen storage for 3 months. (**g**) Changes of PFU activity in cells and supernatants of AdE3-*IAI.3B*-infected A549 carrier cells following liquid nitrogen storage for 3 months.

To formulate A549 carrier cells as cancer gene therapy, drug, radiation, infection, and storage conditions were determined. First, to prevent tumorigenicity of A549 carrier cells in patients, radiation dosage was determined. To evaluate the effect of radiation *in vitro*, A549 cells infected with or without AdE3-*IAI.3B* were irradiated at 200 Gy. A549 cells infected with AdE3-*IAI.3B* at 200 multiplicity of infection (MOI) and irradiated A549 cells infected with or without AdE3-*IAI.3B* at 200 MOI died within 15 days (Figure 2a). To evaluate the effect of radiation *in vivo*, A549 cells were irradiated and subcutaneously inoculated into nude mice, since A549 cells must be prevented from engrafting into the human body because of the possibility that some of A549 carrier cells might not die without infection with oncolytic adenovirus. Irradiation at levels greater than 200 Gy completely suppressed the tumorigenicity of A549 cells in nude mice (Figure 2b).

To determine the infection time of A549 carrier cells, cells were infected with AdE3-*IAI.3B* at 200 MOI with or without antiadenovirus antibodies and the 50% inhibition rate of target ovarian cancer HEY cell growth by A549 carrier cells was calculated. Infection times between 33 and 57 hours showed the most potent antiproliferative effect of AdE3-*IAI.3B*–infected A549 carrier cells with or without antiadenovirus antibodies (*P <* 0.01; Figure 2c). Infection times between 33 and 48 hours showed the most potent antiproliferative effects of A549 carrier cells after freeze-thawing (*P <* 0.01). Carrier cells were preserved in liquid nitrogen to formulate as gene therapy drug and the stability of carrier cells by freeze-store-thawing was examined. However, freeze-thawing rather increased the antiproliferative effects of A549 carrier cells compared with unfrozen carrier cells infected with or without antiadenovirus antibodies between 33 and 57 hours (*P <* 0.05). Since the viability and plaque-forming unit (PFU) activity of A549-*GFP* carrier cells decreased at 48 hours (*P <* 0.05; Figure 2d,e), an incubation time of 33 hours was selected to produce carrier cells. Therefore, A549 carrier cells infected with 200 MOI of AdE3-*IAI.3B* for 33 hours were dissolved in cryopreservative solution of 5% of glycerin and 95% of 5% albumin at 5 × 10^7^ cells/ml, irradiated at 200 Gy, stored in liquid nitrogen until use, and thawed rapidly and injected intratumorally without any processing. Under the liquid nitrogen preservation, the viability of A549 carrier cells and the PFU activity of A549 carrier cells and its supernatant did not change for 3 months (Figure 2f,g). The PFU activity of one A549 carrier cell after freeze-store-thawing was 5 × 10^10^ PFU/5 × 10^7^ cells/ml (Figure 2g).

**Figure 3:**
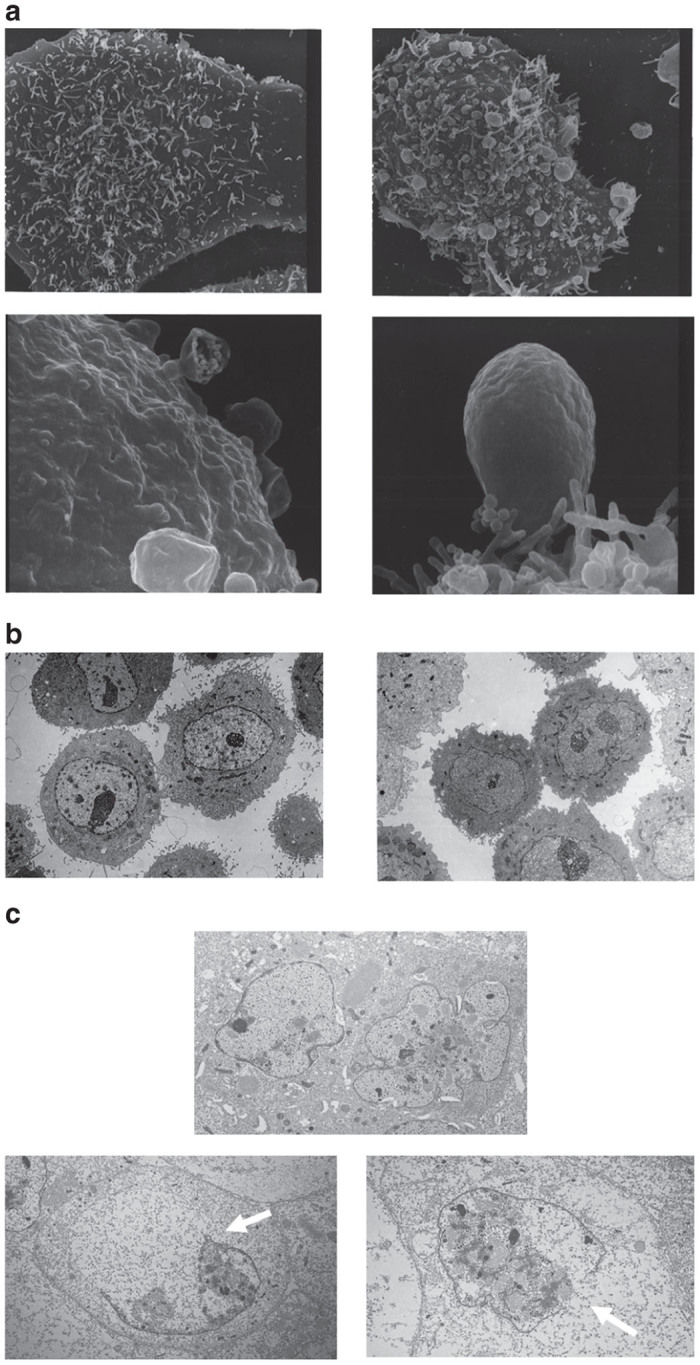
Electron micrographs of AdE3-*IAI.3B*-infected A549 carrier cells. (**a**) Scanning electron micrographs of noninfected A549 cell (upper left, ×4,000), AdE3-*IAI.3B*-infected A549 carrier cell (upper right, ×4,000), and AdE3-*IAI.3B*-infected A549 carrier cell (lower left and right, ×20,000). (**b**) Transmission electron micrographs of noninfected and nonfrozen A549 cells (left, ×3,000), and noninfected and freeze-thawed A549 cells (right, ×3,000). (**c**) Transmission electron micrographs of nonfrozen and AdE3-*IAI.3B*-infected A549 carrier cells (upper ×4,000), and AdE3-*IAI.3B*-infected and freeze-thawed A549 carrier cells (lower left and right; arrows, ruptured nuclear membranes; ×4,000).

To investigate the reason why freeze-thawing increased the antitumor effect of A549 carrier cells, morphological changes were examined using electron microscopy. Scanning electron microscopy demonstrated marked blebbing with released blebs on the surface of A549 carrier cells after 33 hours of infection (Figure 3a). Transmission electron microscopy showed no morphological changes in noninfected A549 cells after freeze-thawing (Figure 3b). However, 33 hours of infection with AdE3-*IAI.3B* at 200 MOI showed multilobulated nuclear membranes in enlarged A549 carrier cells before freezing and ruptured nuclear membranes of further enlarged A549 carrier cells after freeze-thawing (Figure 3c).

### Determination of injection intervals of A549 carrier cells infected with AdE3-*IAI.3B*

**Figure 4:**
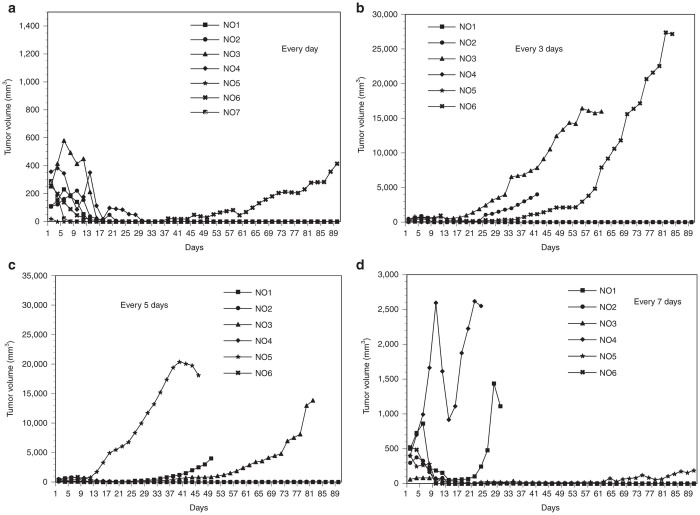
Effects of injection intervals on the antitumor effect of AdE3-*IAI.3B*-infected A549 carrier cells. AdE3-*IAI.3B*-infected A549 carrier cells were injected into OVHM tumors in female mice at the following incidences: every day, *n* = 7 (a), every 3 days, *n* = 6 (b), every 5 days, *n* = 6 (c) and every 7 days, *n* = 6 (d).

To determine the injection intervals of A549 carrier cells, subcutaneous tumors were established in mice using cognate mouse ovarian cancer OVHM cells. Three injections of freeze-thawed A549 carrier cells every 1, 3, 5, and 7 days showed the complete tumor reduction in 6 of 7 mice, 3 of 6 mice, 3 of 6 mice, and 3 of 6 mice, respectively (*P* < 0.05; Figure 4). Therefore, three daily injections of A549 carrier cells were determined to be the most suitable injection protocol of A549 carrier cells for human clinical trials.

### Acute toxicity tests of A549-*GFP* carrier cells infected with AdE3-*IAI.3B* in nude mice

To investigate the toxic properties of A549**-***GFP* carrier cells, human ovarian teratocarcinoma PA-1 cells were injected into the left flanks of female nude mice, and A549**-***GFP* carrier cells were injected into the PA-1 tumors. Intratumoral injections of normal saline, AdE3-*IAI.3B*, A549**-***GFP* cells, and A549**-***GFP* carrier cells infected with AdE3-*IAI.3B* at 200 MOI did not kill any mice (Table 1). Food intake was decreased by the mice injected with AdE3-*IAI.3B* at day 2 and A549 carrier cells at day 2 and 5 (*P <* 0.05) but not in other mice (see Supplementary Table S1). Body weights did not change during the experiment (see Supplementary Table S2). Serum biochemistry tests did not show any abnormalities at day 14 (see Supplementary Table S3).

**Figure 5:**
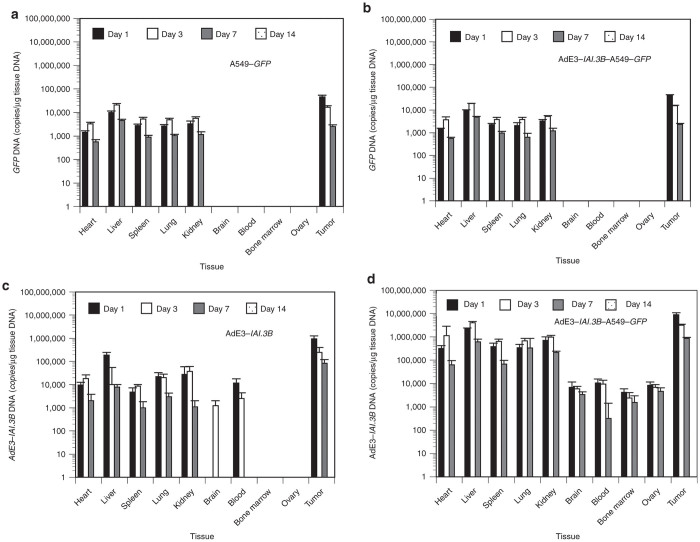
Acute toxicity tests in nude mice. Ten mice per group were sacrificed at 1, 3, 5, 7, and 14 days after injections with A549-*GFP,* AdE3*-IAI.3B* or AdE3-*IAI.3B*-infected A549-*GFP* carrier cells. Organs were excised from each nude mouse and tissue specimens were subjected to quantitative real-time DNA-PCR to quantify *GFP* and AdE3*-IAI.3B* DNA levels. (**a**) DNA content of *GFP* in each organ after injections with A549-*GFP* cells. (**b**) DNA content of *GFP* in each organ after injections with AdE3-*IAI.3B*-infected A549-*GFP* carrier cells. (**c**) DNA content of AdE3-*IAI.3B* in each organ after injections with AdE3-*IAI.3B*. (**d**) DNA content of AdE3-*IAI.3B* after injections of AdE3-*IAI.3B*-infected A549-*GFP* carrier cells.

To determine the body distribution of A549-*GFP* carrier cells, 10 mice per group were sacrificed at 1, 3, 5, 7, and 14 days after injections with A549-*GFP* cells, AdE3-*IAI.3B*, and AdE3-*IAI.3B*–infected A549-*GFP* carrier cells, and excised tissue was subjected to quantitative real-time DNA-PCR analysis for *GFP* and AdE3-*IAI.3B*. *GFP* DNA peaked at day 1 in tumors and at day 3 in the liver, heart, spleen, lungs, and kidneys and was expressed until day 7 but was no longer apparent at day 14. *GFP* DNA was not expressed in the brain, blood, bone marrow, or ovarian tissue during the experiment (Figure 5a). *GFP* DNA expression after the injection of A549-*GFP* carrier cells was similar to those of A549-*GFP* cells (Figure 5b). In the AdE3-*IAI.3B*–injected mice, AdE3-*IAI.3B* DNA in the tumor, liver, lungs, and blood peaked at day 1 and that in heart, spleen, and kidneys peaked at day 3 and was expressed until day 7 but was not evident at day 14. AdE3-*IAI.3B* DNA was detected in the brain at day 3 and in the blood at days 1 and 3. Bone marrow and ovarian tissue did not express AdE3-*IAI.3B* DNA (Figure 5c). In A549-*GFP* carrier cell–injected mice, AdE3-*IAI.3B* DNA peaked at the highest level overall at day 1 in the tumor and at day 3 in heart, liver, spleen, lungs, and kidneys and also peaked at day 1 in brain, blood, bone marrow, and ovary. Expression was observed at day 7 but not at day 14. The DNA content of AdE3-*IAI.3B* in the tumor, heart, liver, spleen, lungs, and kidneys was one or two orders higher than those after the injections of AdE3-*IAI.3B* (*P* < 0.05; Figure 5d).

### Chronic toxicity tests of A549 carrier cells infected with AdE3-*IAI.3B* in rabbits

To determine the chronic toxicity of AdE3-*IAI.3B*–infected A549 carrier cells, A549 cells, AdE3-*IAI.3*, and A549 carrier cells infected with AdE3-*IAI.3B* at 200 MOI were injected subcutaneously into female rabbits. As shown in Table 2, five of 10 rabbits injected with a high dose of A549 carrier cells died; four in the first 4 weeks of the experiments and the other in the subsequent 4 weeks of follow-up. One of 10 rabbits injected with A549 cells died in the first 4 weeks, and no rabbits injected with AdE3-*IAI.3B*, or low and moderate doses of A549 carrier cells, died. The histopathological findings of the six dead rabbits revealed lysis of the intestine wall, pancreas, and spleen after injections with A549 cells and A549 carrier cells; necrosis of the liver and injected site; inflammation of lungs and appendix; and splenomegaly only after injections with A549 carrier cells (see Supplementary Table S4). From these results, less than the moderate dose of 1.25 × 10^7^ cells/kg A549 carrier cells, which is 40 times greater than that of estimated standard human clinical use, is suggested to be recommended in human clinical trials. Skin lesions of the injection site were detected in rabbits at 24 hours after the final injection of carrier cells. Splenomegaly was not detected in A549 cell–injected rabbits but was detected in the majority of A549 carrier cell–injected rabbits even at 4 weeks after the final injections (see Supplementary Tables S5 and S6). The splenomegaly seemed to be a result of immunoreaction against the oncolytic adenovirus of injected A549 carrier cells, since the size of the spleen was related to antiadenovirus antibody titers (*P* < 0.05).

In hematology tests, white blood cell and neutrophil counts were increased at 2 weeks after the first injections of any dose of carrier cells, but hemoglobin was decreased at 4 weeks after the first injections (*P* < 0.05). Red blood cell, hemoglobin, and platelet counts were decreased at 2 and 4 weeks after the first injections of the high-dose carrier cells (*P* < 0.05; see Supplementary Table S7). Hemostatic tests revealed that fibrinogen and thrombin time were increased at 4 weeks after the first injections of A549 cells and any dose of carrier cells (*P* < 0.05; see Supplementary Table S8). Biochemical tests showed alanine aminotransferase, alkaline phosphatase, gamma-glutamyl transpeptidase, and albumin were decreased, but total protein was increased at 4 weeks after the first injections of any dose of carrier cells (*P* < 0.05; see Supplementary Table S9). Triglyceride and total cholesterol were increased at 2 weeks after the first injections of high dose of carrier cells (*P* < 0.05; see Supplementary Table S10).

**Figure 6:**
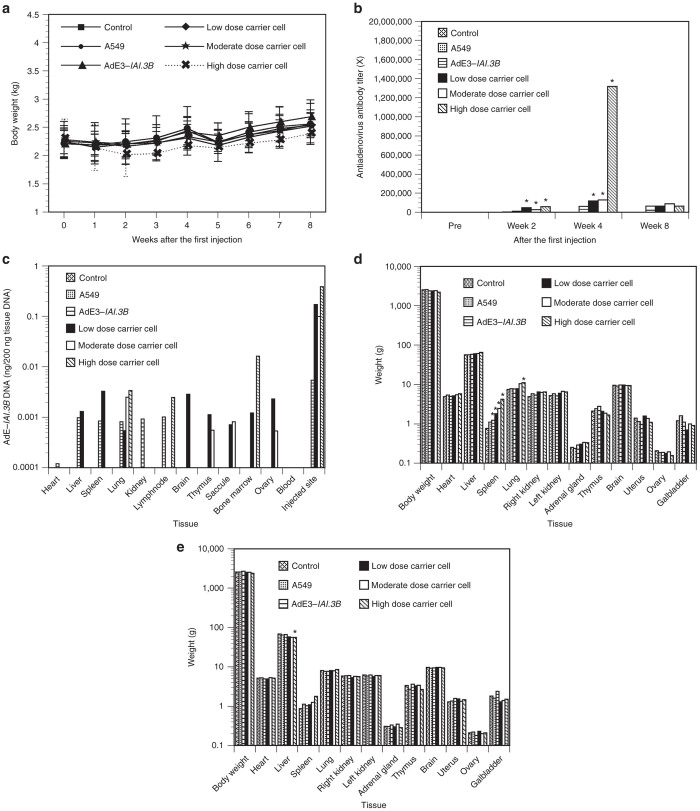
Chronic toxicity tests in rabbits. A549 cells, AdE3-*IAI.3B*, and low, medium and high dose of AdE3-*IAI.3B*-infected A549 carrier cells were subcutaneously administered to each rabbit on eight occasions over a 4 week periods. (**a**) Changes in body weight. (**b**) Changes in antiadenovirus antibody titers. (**c**) DNA content of AdE3-*IAI.3B* at 24 hours after the final injections with AdE3-*IAI.3B*-infected A549 carrier cells. (**d**) Weight of each organ at 24 hours after the final injections with AdE3-*IAI.3B*-infected A549 carrier cells (each dose without high dosage of carrier cells, *n* = 6; high dosage carrier cells, *n* = 2). (**e**) Weight of each organ at 4 weeks after the final injections with AdE3-*IAI.3B*-infected A549 carrier cells (each dose without high dose of carrier cells, *n* = 4; high dose carrier cells, *n* = 3). *P < 0.05.

Body weight was decreased at 3 and 4 weeks after the first injections of high dose of carrier cells (*P* < 0.05; Figure 6a). The antiadenovirus antibodies in AdE3-*IAI.3B*– and carrier cell–injected rabbits were increased from the second week, peaked at the fourth week then decreased, and the difference between the groups had disappeared at the eighth week (*P* < 0.05; Figure 6b). The antiadenovirus antibody titers at 2 and 4 weeks after the first injections of low and moderate dose of carrier cells were 2–4 times those after the first injections of AdE3-*IAI.3B*, and those at 2 and 4 weeks after the first injections of high dose of carrier cells were 5 and 21 times those after the first injections of AdE3-*IAI.3B*, respectively (*P* < 0.05). AdE3-*IAI.3B* DNA at 24 hours after the final injections was detected in all tissues except blood and was at the highest levels in the injected site (Figure 6c). The DNA content of AdE3-*IAI.3B* in the injected site at 24 hours after the final injections of low, moderate, and high dose of carrier cells was 30, 20, and 70 times that after the final injections of AdE3-*IAI.3B*, respectively (*P* < 0.05). The weight of the spleen at 24 hours after the final injections was increased in order of increasing of dose of carrier cells (*P* < 0.05; Figure 6d). Liver weight alone was decreased at 4 weeks after the final injections of high dose carrier cells (*P* < 0.05; Figure 6e).

## Discussion

This study revealed that IAI.3B promoter was most active in ovarian cancer cells and also in other cancer cells but not in normal cells. *IAI.3B* promoter–driven oncolytic adenovirus, *AdE3-IAI.3B*, selectively killed ovarian cancer cells in particular, along with other cancer cell types, but not normal cells. In contrast, wild-type adenovirus, AdE3, killed both cancer and normal cells. Furthermore, AdE3-*IAI.3B*–infected A549 carrier cells killed ovarian and other cancer cells but not normal cells. The tissue specificity of *IAI.3B* promoter and AdE3-*IAI.3B* for ovarian cancer was reported previously,^22^ but specificity for cervical cancer, skin cancer, esophageal cancer, head and neck cancer, glioma, lung cancer, stomach cancer, colon cancer, pancreatic cancer, liver cancer, prostate cancer, breast cancer, mesothelioma, and neurofibromatosis type 1 (NF1) cells has not yet been reported. A wide range of tumor-specific markers and promoters are well known for *midkine and cyclooxygenase-2*, and these promoter-driven oncolytic adenoviruses kill various types of cancers.^23–26^ As for the results in this study, it is clear that oncolytic adenovirus driven by *IAI.3B* promoter has a very wide antitumor spectrum for solid tumors, similar to the oncolytic adenovirus vectors driven by the *cyclooxygenase-2* and *midkine* promoters.

Irradiation at 200 Gy completely inhibited *in vitro* cell proliferation and *in vivo* tumorigenicity of A549 cells. Furthermore, the ability of A549 cells to proliferate *in vitro* was completely inhibited by infection with AdE3-*IAI.3B* at 200 MOI. However, it might be considered that 200 Gy of irradiation should be used together with AdE3-*IAI.3B* infection for safer clinical trials, since A549 carrier cells may survive by chance because of insufficient adenoviral infection of some cells.

Scanning electron microscopy demonstrated that the infection of AdE3-*IAI.3B* at 200 MOI for 33 hours resulted in blebbing on the surface of the carrier cells, as previously observed by transmission electron microscopy.^13^ Furthermore, transmission electron microscopy revealed lobulated nuclear membranes. Adenovirus death protein is a transmembrane protein that lyses nuclear and cellular membranes and results in cell death. The lobulated nuclear membranes and blebbing of cell surfaces might be due to fragile nuclear and cellular membranes induced by adenovirus death protein. Freeze-thawing increased the antitumor activity of carrier cells with or without neutralizing antibodies. Transmission electron microscopy demonstrated that lobulated nuclear membranes ruptured after freeze-thawing and showed a number of adenovirus particles moved to the cytoplasm from the ruptured nuclei. Thus, the increased adenovirus particles in the cytoplasm may move into the formed blebs on the cell surface, and the amount of adenovirus particles in the blebs may relatively increase. Therefore, the procedure of freeze-thawing might increase the antitumor activity of carrier cells by increasing the oncolytic adenovirus content in the cytoplasm and the blebs.

Three consecutive daily intratumoral injections demonstrated the most potent antitumor activity, which resulted in the complete tumor reduction in 6 of 7 mice. DNA-PCR analysis demonstrated that the DNA content of AdE3-*IAI.3B* and A549-*GFP* was the highest in the tumors at one day after injections with AdE3-*IAI.3B*–infected A549-*GFP* carrier cells and decreased to one-third and one-tenth to twentieth at days 3 and 7 after the injections, respectively. Thus, daily injections seem to be necessary for a human clinical trial because the content of oncolytic adenovirus in the tumor decreases rapidly at days 3 and 7 after injections of carrier cells. The adenoviral clearance rate from the tumor was not significantly different between the injections with oncolytic adenovirus and carrier cells, but the intratumoral oncolytic adenoviral content after carrier cell injection was one order higher than that after injections with oncolytic adenovirus. This high oncolytic adenovirus content in the tumor might be a main cause of the significant antitumor activity of carrier cell. The DNA content of AdE3-*IAI.3B* after carrier cell injections in nontumor organs was also one or two order higher than that after AdE3-*IAI.3B* injections. This may indicate that oncolytic adenovirus is distributed at high concentrations throughout almost the whole body after carrier cell injections, and carrier cell treatment is effective not only for the injected target tumors but also for systemically metastasized tumors.

One rabbit died within the first 4 weeks following injections with A549 cells, and postmortem assessment showed lysis of the pancreas, intestinal wall, and spleen. A549-injected rabbits had increased fibrinogen and thrombin time, which might indicate an initial state of hypercoagulability and hyperfibrinolysis, respectively, although this is not as severe as disseminated intravascular coagulation. Therefore, injection of a number of A549 cells might cause mild hypercoagulability and hyperfibrinolysis caused by A549 cell death and result in a serious organ disorder in a dead rabbit. After injections with a high dose of carrier cells, 4 of 10 rabbits died in the first 4 weeks following injections, and 1 died in the next 4 weeks. Body weight had decreased by 3 and 4 weeks after the first injections. Therefore, it can be concluded that high doses of carrier cells resulted in serious or fatal side effects and that less than a moderate dose should be provided in human clinical trials. The cell numbers of high dose of carrier cells were 100–200 times those of high dose of GVAX tumor vaccine clinical trials,^27,28^ which were less than those of low dose and consistent with those of high dose of the scheduled clinical trial of carrier cells. Carrier cells infected with adenovirus-GM-CSF and oncolytic adenovirus could be expected to show a potent antitumor activity compared with GM-CSF expressing GVAX and to carry out a safe clinical trial because of no severe side effects in less than moderate dose of carrier cells.

AdE3-*IAI.3B* DNA at 24 hours after the final injections with AdE3-*IAI.3B* and carrier cells was detected in all organs except the blood, but this was not dose-dependent except at the injection site at which there were the highest levels of high dose carrier cells and the lowest of AdE3-*IAI.3B*. The weight of the spleen increased in a dose-dependent manner after injections with AdE3-*IAI.3B* and carrier cells at 24 hours after the final injections. Since the weight of the spleen is correlated with the value of neutralizing antibodies, the difference between spleen weights is thought to be due to an immune response against adenovirus.

Total cholesterol and triglyceride increased by twofold at 2 weeks after the first injections of high-dose carrier cells. It has been reported that adenovirus infection is associated with adiposity status and increases total cholesterol and triglyceride.^29^ Alanine aminotransferase, alkaline phosphatase, gamma-glutamyl transpeptidase, liver enzyme, and albumin were decreased after injections with any dose of carrier cells. This might indicate that these proteins decreased due to the inhibition of the production in the liver as a result of damage by high dose of oncolytic adenovirus and lysis of carrier cells or by the binding of these proteins to the increased immunoglobulin. The increase of total protein despite the reduction in albumin might be due to the increase in immunoglobulin, as evidenced by the increased spleen weights and antiadenovirus antibodies after injections with carrier cells.

White blood cell and neutrophil counts, and fibrinogen levels and thrombin time were increased, and hemoglobin and platelet counts were decreased after injections of carrier cells. Fibrinogen might be increased by the acute inflammatory reaction caused by necrosis and lysis of carrier cells to induce microvascular hypercoagulability. Furthermore, this microvascular hypercoagulability might cause hemolysis, which decreases hemoglobin and platelet levels, and elongates thrombin time. Since fibrinogen and thrombin time also increased after injections with A549 cells but not with oncolytic adenovirus, these might be caused by necrosis and lysis of the injected cells. In addition, these changes may result from compensatory disseminated intravascular coagulation, because prothrombin time and activated partial thromboplastin time did not change.

Although the DNA of A549-*GFP* cells and oncolytic adenovirus accumulated in the liver after carrier cell injections, liver function was slightly suppressed but not severely damaged without the elevation of liver enzymes. This might indicate that a considerable proportion of adenovirus in the liver did not have bioactivity, and the oncolytic adenovirus AdE3-*IAI.3B* was not so amplified in the liver because of the tumor specificity of the *IAI.3B* promoter, because liver enzymes were not increased in any rabbits injected not only with AdE3-*IAI.3B* but also with carrier cells. Electron microscopy demonstrated that carrier cells had a number of blebs on their surface, which contained the adenoviral particles, and the infection inhibition of oncolytic adenovirus by neutralizing antibodies might be evaded by isolating the blebs from the cell surface. Furthermore, the freeze-thaw procedure enhanced the antitumor activity of the carrier cells by breaking down the nuclear membrane and releasing adenovirus particles from the nucleus to the cytoplasm to significantly reduce tumor growth even after the preimmunization to adenovirus without the combination of adenovirus-*GM-CSF*. The carrier cell therapy also induced a significant immune response based on the evidence of the increased antiadenovirus antibodies and splenomegaly. This might indicate that carrier cells induce not only humoral immunity but also antiadenoviral and antitumor cellular immunity that is advantageous for the induction of cytotoxic T lymphocytes to treat tumors.

## Materials and Methods

### Cell lines and adenoviruses

Human ovarian cancer HEY, PA-1, RMG-1, 420, OCCI, OVCAR3, KK, KF, 429, DOV13, and MH cells, human cervical carcinoma SKGIIIa, HT-III, CaSki, and HeLa cells, human skin cancer HCS-5 cells, human esophageal cancer ECG1-10 cells, human head and neck cancer HSC4, HSC3, HSC2, and Ca-9–22 cells, human glioma U373 and U251 cells, human non–small-cell lung cancer A549 and H1299 cells, human stomach cancer AGS cells, human colon cancer HT29 and SW626 cells, human pancreatic cancer Panc I cells, human hepatocellular carcinoma HepG2 cells, human prostate cancer LNCap, C4-2B, and CWR22rv cells, human breast cancer T47D, MCF-7, and BT-549 cells, human mesothelioma 211H, H29, H226, and H2452 cells, human neurofibromatosis type 1 NF1 NF2, NF3-1, NF3-2, and NF3-3 cells, human normal ovarian fibroblast NOE-1, NOE-2, and NOE-3 cells, keratinocyte K42 cells, skin fibroblast F27 cells, umbilical vein endothelial HUVEC cells, and murine ovarian cancer OVHM cells were cultured as described previously.^13,22^ Construction, purification, and PFU assay of adenoviruses were performed as described previously.^22^ PFU activity was determined for A549 carrier cells during infection with AdE3-*IAI.3B*, and cells and supernatant of A549 carrier cells after thawing following liquid nitrogen storage.

### Assay for *IAI.3B* promoter activity

*IAI.3B* promoter consisting of 1,875 bp was inserted into the luciferase reporter vector PicaGene Basic, a promoterless and enhancerless vector (Toyo Ink MFG, Tokyo, Japan) and was transfected into cells in the presence of N-[1-(2,3-dioleoyloxyl)propyl]-N,N,N-trimethylammoniummethyl sulfate liposomal transfection reagent (Roche Molecular Biochemicals, Indianapolis, IN). Dual luciferase assays were performed according to the manufacturer’s protocol (Promega, Tokyo, Japan).

### Cell count assay

To determine the cytotoxic effects of AdE3-*IAI.3B*, AdE3, and A549 carrier cells on each cell line, cells were plated at a density of 10,000 cells/well in 12-well plates, cultured for 48 hours with AdE3-*IAI.3B*, AdE3, or A549 carrier cells infected with AdE3-*IAI.3B* or AdE3 at 200 MOI for 16 hours, and then counted. To determine the effect of antiadenovirus antibodies and freeze-thawing on the cytotoxicity of A549 carrier cells, ovarian cancer HEY cells were incubated for 5 days with A549 carrier cells infected for 12–72 hours with AdE3-*IAI.3B* at 200 MOI with or without antiadenovirus antibodies (Takeda Pharmaceutical, Tokyo, Japan) and with or without freeze-thawing of A549 carrier cells. Cell viability of A549 carrier cells before and after freeze-thawing was determined by trypan blue-exclusion test.

### Electron microscopy

A549 cells were infected with AdE3-*IAI.3B* at 200 MOI for 33 hours, frozen, stored in liquid nitrogen, and thawed rapidly at 37 °C, then fixed with 2% glutaraldehyde, and scraped. Carrier cells were further fixed with 2% buffered-osmium tetroxide for 2 hours, examined by scanning electron microscopy, embedded in Epon epoxy resin, stained with uranyl acetate and lead citrate, and examined by transmission electron microscopy.

### Inhibition of subcutaneous ovarian tumor growth in syngeneic mice

Murine ovarian carcinoma OVHM cells were injected into female (C57BL/6 × C3H/He) F1 mice to form subcutaneous tumors after the preimmunization with Ad-*β gal* at 1 × 10^10^ PFU/mouse. A549 carrier cells were infected with AdE3-*IAI.3B* at 200 MOI for 33 hours, irradiated at 200 Gy, frozen, and stored in liquid nitrogen, then rapidly thawed at 37 °C. These cells were then injected three times into tumors of 5–8 mm in diameter every 1, 3, 5, and 7 days. Tumor volume was calculated by assuming a spherical shape, with the average tumor diameter calculated as the square root of the product of cross-sectional diameters.

### Acute toxicity tests

PA-1 cells (1 × 10^7^) were injected into female nude (*nu/nu*) mice (*n* = 10). Then, 0.2 ml of control saline, A549-*GFP* cells (1 × 10^7^ cells), AdE3-*IAI.3B* (2 × 10^9^ PFU), or A549-*GFP* carrier cells (1 × 10^7^ cells) infected with AdE3-*IAI.3B* at 200 MOI for 33 hours were injected into tumors of 5–8 mm in diameter. Any changes in the amount of food intake and body weight were observed, and blood was collected at day 14 to examine serum biochemistry. Ten mice per each group were sacrificed at 1, 3, 5, 7, and 14 days after the injections with A549-*GFP* cells, AdE3-*IAI.3B*, and AdE3-*IAI.3B*–infected A549-*GFP* carrier cells and subjected to quantitative real-time DNA-PCR analysis for *GFP* and AdE3-*IAI.3B*.

### Chronic toxicity tests

Control saline, A549 cells (5 × 10^7^ cells/kg), AdE3-*IAI.3B* (1 × 10^10^ PFU/kg) and low (2.5 × 10^6^ cells/kg), moderate (1.25 × 10^7^ cells/kg), and high (5 × 10^7^ cells/kg) doses of A549 carrier cells infected with AdE3-*IAI.3B* for 33 hours at 200 MOI were subcutaneously injected eight times over 4 weeks into female New Zealand white rabbits (*n* = 10 pre group), which could be infected by adenovirus,^30^ and observed for an additional 4-week period. Each rabbit was subjected to hematological examination 2 days before and 2, 4, and 8 weeks after the first injections. Autopsies were performed on six rabbits in each group at 24 hours after the final injections, and on all dead rabbits and the remaining rabbits in each group at 4 weeks after the final injections. Neutralizing antiadenovirus antibodies and anti-A549 antibodies were measured as previously described.^13,31^ Anti-A549 antibodies were not detected in any rabbits.

### Statistical analysis

Values are expressed as the mean ± SD and were analyzed with the unpaired *t*-test, Welch test, and regression analysis. Survival data were analyzed with the generalized Wilcoxon test. Statistical significance was set at *P <* 0.05.

## Figures and Tables

**Table 1:** Protocol and survival rates of acute toxicity tests in nude mice

*Treatment*	*Volume*	*Dose*	*Relative dose*	*Survival*
Saline	0.2 ml	—	—	10/10
AdE3-*IAI.3B*	0.2 ml	2 × 10^9^ PFU	—	10/10
A549-*GFP*	0.2 ml	1 × 10^7^ cells	—	10/10
Carrier cells	0.2 ml	1 × 10^7^ cells	3,000 times	10/10

AdE3-*IAI.3B*, A549-*GFP* cells, and carrier cells (AdE3-*IAI.3B*-infected A549-*GFP*) were injected into subcutaneous ovarian PA-1 tumors in nude mice. Relative doses were calculated as 20 g and 60 kg of mouse and human body weights, respectively.

**Table 2:** Protocol and survival rates of chronic toxicity tests in rabbits

*Treatment*	*Dose (8 times/4 week)*	*Relative dose*	*Survival*
Control	—	—	10/10
A549 cell	5 × 10^7^ cells/kg	—	9/10
AdE3-*IAI.3B*	1 × 10^10^ PFU/kg	—	10/10
Low dose carrier cells	2.5 × 10^6^ cells/kg	8 times	10/10
Moderate dose carrier cells	1.25 × 10^7^ cells/kg	40 times	10/10
High dose carrier cells	5 × 10^7^ cells/kg	160 times	5/10

Carrier cells: AdE3-*IAI.3B*–infected A549 cells. Relative dose: relative ratio to average treatment dose in humans. Relative doses were calculated as 2 kg and 60 kg of rabbit and human body weights, respectively.

## References

[bib1] Geoerger B Grill J Opolon P Morizet J Aubert G Terrier-Lacombe MJ 2002 Oncolytic activity of the E1B-55 kDa-deleted adenovirus ONYX-015 is independent of cellular p53 status in human malignant glioma xenografts Cancer Res 62 764 772 11830531

[bib2] DeMatteo RP Yeh H Friscia M Caparrelli D Burke C Desai N 1999 Cellular immunity delimits adenoviral gene therapy strategies for the treatment of neoplastic diseases Ann Surg Oncol 6 88 94 10030420 10.1007/s10434-999-0088-2

[bib3] Mok H Palmer DJ Ng P Barry MA 2005 Evaluation of polyethylene glycol modification of first-generation and helper-dependent adenoviral vectors to reduce innate immune responses Mol Ther 11 66 79 15585407 10.1016/j.ymthe.2004.09.015

[bib4] Steel JC Cavanagh HM Burton MA Kalle WH 2004 Microsphere-liposome complexes protect adenoviral vectors from neutralising antibody without losses in transfection efficiency, in-vitro J Pharm Pharmacol 56 1371 1378 15525443 10.1211/0022357044643

[bib5] Jooss K Yang Y Wilson JM 1996 Cyclophosphamide diminishes inflammation and prolongs transgene expression following delivery of adenoviral vectors to mouse liver and lung Hum Gene Ther 7 1555 1566 8864756 10.1089/hum.1996.7.13-1555

[bib6] Bouvet M Fang B Ekmekcioglu S Ji L Bucana CD Hamada K 1998 Suppression of the immune response to an adenovirus vector and enhancement of intratumoral transgene expression by low-dose etoposide Gene Ther 5 189 195 9578838 10.1038/sj.gt.3300564

[bib7] Coukos G Makrigiannakis A Kang EH Caparelli D Benjamin I Kaiser LR 1999 Use of carrier cells to deliver a replication-selective herpes simplex virus-1 mutant for the intraperitoneal therapy of epithelial ovarian cancer Clin Cancer Res 5 1523 1537 10389942

[bib8] Hakkarainen T Särkioja M Lehenkari P Miettinen S Ylikomi T Suuronen R 2007 Human mesenchymal stem cells lack tumor tropism but enhance the antitumor activity of oncolytic adenoviruses in orthotopic lung and breast tumors Hum Gene Ther 18 627 641 17604566 10.1089/hum.2007.034

[bib9] Munguia A Ota T Miest T Russell SJ 2008 Cell carriers to deliver oncolytic viruses to sites of myeloma tumor growth Gene Ther 15 797 806 18356812 10.1038/gt.2008.45

[bib10] Thorne SH Negrin RS Contag CH 2006 Synergistic antitumor effects of immune cell-viral biotherapy Science 311 1780 1784 16556847 10.1126/science.1121411

[bib11] Raykov Z Balboni G Aprahamian M Rommelaere J 2004 Carrier cell-mediated delivery of oncolytic parvoviruses for targeting metastases Int J Cancer 109 742 749 14999784 10.1002/ijc.20013

[bib12] Qiao J Kottke T Willmon C Galivo F Wongthida P Diaz RM 2008 Purging metastases in lymphoid organs using a combination of antigen-nonspecific adoptive T cell therapy, oncolytic virotherapy and immunotherapy Nat Med 14 37 44 18066076 10.1038/nm1681

[bib13] Hamada K Desaki J Nakagawa K Zhang T Shirakawa T Gotoh A 2007 Carrier cell-mediated delivery of a replication-competent adenovirus for cancer gene therapy Mol Ther 15 1121 1128 17387337 10.1038/sj.mt.6300128

[bib14] Perrotte P Wood M Slaton JW Wilson DR Pagliaro L Price RE 2000 Biosafety of *in vivo* adenovirus-p53 intravesical administration in mice Urology 56 155 159 10869658 10.1016/s0090-4295(00)00537-9

[bib15] Huang BJ Liu RY Huang JL Liang ZH Gao GF Wu JX 2007 Long-term toxicity studies in Canine of E10A, an adenoviral vector for human endostatin gene Hum Gene Ther 18 207 221 17346097 10.1089/hum.2006.149

[bib16] Li Y Shao JY Liu RY Zhou L Chai LP Li HL 2008 Evaluation of long-term toxicity of Ad/hIFN-, an adenoviral vector encoding the human interferon-gamma gene, in nonhuman primates Hum Gene Ther 19 827 839 18666838 10.1089/hum.2007.180

[bib17] Engler H Machemer T Philopena J Wen SF Quijano E Ramachandra M 2004 Acute hepatotoxicity of oncolytic adenoviruses in mouse models is associated with expression of wild-type E1a and induction of TNF-alpha Virology 328 52 61 15380358 10.1016/j.virol.2004.06.043

[bib18] Su C Cao H Tan S Huang Y Jia X Jiang L 2008 Toxicology profiles of a novel p53-armed replication-competent oncolytic adenovirus in rodents, felids, and nonhuman primates Toxicol Sci 106 242 250 18703561 10.1093/toxsci/kfn168

[bib19] Nemunaitis J Sterman D Jablons D Smith JW 2nd Fox B Maples P 2004 Granulocyte-macrophage colony-stimulating factor gene-modified autologous tumor vaccines in non-small-cell lung cancer J Natl Cancer Inst 96 326 331 14970281 10.1093/jnci/djh028

[bib20] Thaci B Ahmed AU Ulasov IV Tobias AL Han Y Aboody KS 2012 Pharmacokinetic study of neural stem cell-based cell carrier for oncolytic virotherapy: targeted delivery of the therapeutic payload in an orthotopic brain tumor model Cancer Gene Ther 19 431 442 22555507 10.1038/cgt.2012.21 PMC3356460

[bib21] Campbell IG Nicolai HM Foulkes WD Senger G Stamp GW Allan G 1994 A novel gene encoding a B-box protein within the BRCA1 region at 17q21.1 Hum Mol Genet 3 589 594 8069304 10.1093/hmg/3.4.589

[bib22] Hamada K Kohno S Iwamoto M Yokota H Okada M Tagawa M 2003 Identification of the human IAI.3B promoter element and its use in the construction of a replication-selective adenovirus for ovarian cancer therapy Cancer Res 63 2506 2512 12750273

[bib23] Tanaka H Shirakawa T Zhang Z Hamada K Gotoh A Nibu K 2005 A replication-selective adenoviral vector for head and neck cancers Arch Otolaryngol Head Neck Surg 131 630 634 16027288 10.1001/archotol.131.7.630

[bib24] Shirakawa T Hamada K Zhang Z Okada H Tagawa M Kamidono S 2004 A cox-2 promoter-based replication-selective adenoviral vector to target the cox-2-expressing human bladder cancer cells Clin Cancer Res 10 4342 4348 15240520 10.1158/1078-0432.CCR-03-0267

[bib25] Kohno S Nakagawa K Hamada K Harada H Yamasaki K Hashimoto K 2004 Midkine promoter-based conditionally replicative adenovirus for malignant glioma therapy Oncol Rep 12 73 78 15201962

[bib26] Terao S Shirakawa T Kubo S Bishunu A Lee SJ Goda K 2007 Midkine promoter-based conditionally replicative adenovirus for targeting midkine-expressing human bladder cancer model Urology 70 1009 1013 17919690 10.1016/j.urology.2007.07.003

[bib27] Na HN Hong YM Kim J Kim HK Jo I Nam JH 2010 Association between human adenovirus-36 and lipid disorders in Korean schoolchildren Int J Obes (Lond) 34 89 93 19823186 10.1038/ijo.2009.207

[bib28] Tani K Azuma M Nakazaki Y Oyaizu N Hase H Ohata J 2004 Phase I study of autologous tumor vaccines transduced with the GM-CSF gene in four patients with stage IV renal cell cancer in Japan: clinical and immunological findings Mol Ther 10 799 816 15451464 10.1016/j.ymthe.2004.07.001

[bib29] Simons JW Carducci MA Mikhak B Lim M Biedrzycki B Borellini F 2006 Phase I/II trial of an allogeneic cellular immunotherapy in hormone-naïve prostate cancer Clin Cancer Res 12 11 Pt 1 3394 3401 16740763 10.1158/1078-0432.CCR-06-0145

[bib30] Tancevski I Frank S Massoner P Stanzl U Schgoer W Wehinger A 2005 Increased plasma levels of LDL cholesterol in rabbits after adenoviral overexpression of human scavenger receptor class B type I J Mol Med 83 927 932 16133421 10.1007/s00109-005-0695-8

[bib31] Sarkar AK Mitchell MF Hamada K Buchl SJ Satterfield WC Schapiro SJ 1999 Evaluation of cellular immune responses in rhesus monkeys subjected to adenovirus-mediated gene transfer into the cervix Cancer Gene Ther 6 220 227 10359207 10.1038/sj.cgt.7700046

