# Supplementary material for: Biosafety studies of carrier cells infected with a replication-competent adenovirus introduced by IAI.3B promoter
Source: Mol Ther Methods Clin Dev. 2014 May 28;1:14019–. doi: 10.1038/mtm.2014.19 (PMC4362384; doi:10.1038/mtm.2014.19)
Supplement: Supplementary Tables [file mtm201419-s1.doc]

**SUPPLEMENTARY INFORMATION**

Table S1. Food intake changes (g) of nude mice

|  |
| --- |

Date Saline AdE3-*IAI.3B* A549 Carrier cells

|  |
| --- |

Day 2 5.2±0.2 2.8±0.2* 4.8±0.2 2.3±0.2*

Day 5 3.9±0.1 3.7±0.2 4.0±0.2 3.2±0.3*

Day 8 3.4±0.1 3.1±0.1 3.5±0.1 3.6±0.3

Day 11 3.2±0.2 3.4±0.1 3.4±0.1 3.3±0.1

Day 15 3.6±0.1 3.4±0.1 3.8±0.2 3.5±0.35

|  |
| --- |

AdE3-*IAI.3B*, A549*-GFP* cells and carrier cells (AdE3-*IAI.3B*-infected A549-*GFP*) were injected into subcutaneous ovarian PA-1 tumors in nude mice. *, *P* < 0.05 Values represent the means ± SD.

Table S2. Changes of body weight (g) of nude mice

|  |
| --- |

Date Saline AdE3-*IAI.3B* A549 Carrier cells

|  |
| --- |

Day 0 15.5±0.8 15.6±0.9 15.5±0.7 15.5±0.8

Day 2 16.5±1.1 15.5±1.0 16.7±0.7 15.4±0.8

Day 5 17.4±1.1 16.9±0.9 17.4±0.6 16.5±0.5

Day 8 17.8±0.9 17.0±1.0 17.8±0.7 17.4±0.5

Day 11 17.8±0.7 17.6±1.0 18.0±0.6 17.6±0.6

Day 15 18.5±0.8 18.4±0.9 18.7±0.8 18.4±0.6

Weight gain 3.0±0.8 2.8±0.8 3.3±0.9 3.0±0.6

|  |
| --- |

AdE3-*IAI.3B*, A549 cells and carrier cells (AdE3-*IAI.3B*-infected A549-*GFP*) were injected into subcutaneous ovarian PA-1 tumors in nude mice. Values represent the means ± SD.

Table S3. Results of serum biochemistry tests in nude mice

|  |
| --- |

Serum chemistry Saline AdE3-*IAI.3B* A549 Carrier cells

|  |
| --- |

ALT (U/L) 65±12 63±19 48±8 52±8

AST (U/L) 180±47 166±40 131±25 125±27

BUN (mmol/L) 10.5±1.9 8.7±1.4 8.63±1.91 7.89±2.12

Creatine (uml/L) 33.5±5.9 34.1±13.6 41.1±8.3 30.4±11.7

|  |
| --- |

AdE3-*IAI.3B*, A549 cells and carrier cells (AdE3-*IAI.3B*-infected A549-*GFP*) were injected

into subcutaneous ovarian PA-1 tumors in nude mice. Values represent the means ± SD.

Table S4. Results of histopathological tests of dead rabbits following chronic toxicity tests

|  |
| --- |

Pathological finding Mild Moderate Severe

|  |
| --- |

Liver necrosis H8 --- ---

Lysis of pancreas B2, H8 --- ---

Lysis of intestine wall B2, H8, H10 --- ---

Lysis of spleen B2, H8 --- ---

Splenomegaly H5 H9 ---

Necrosis of injected site H3, H8, H9, H10 --- ---

Interstitial pneumonitis --- H9 ---

Interstitial appendicitis H5 --- ---

|  |
| --- |

B: A549 cell group, H: high dose carrier cell group. The number next to B and H indicates the

individual number of each rabbit.

Table S5. Results of histopathological tests at 24 h after the final injections of carrier cells following chronic toxicity tests in rabbits

|  |
| --- |

Control A549 AdE3-*IAI.3B* Carrier cells (dose)

low moderate high

N 6 5 6 6 6 2

|  |
| --- |

Skin necrosis ----- + (2) + (1) + (6) + (6) + (2)

Lung hemorrhage ----- + (1) ----- ----- ----- -----

Liver cirrhosis ----- ----- ----- ----- ----- -----

Liver necrosis ----- ----- ----- ----- ----- -----

Liver edema ----- + (1) + (1) + (1) + (1) -----

Liver inflammation ----- ----- ----- ----- ----- + (1)

Splenomegaly ----- ----- + (5) ++ (6) ++ (6) +++ (2)

Gallbladder mucosal ----- ----- ----- ----- ----- + (1)

necrosis

|  |
| --- |

Number in parentheses indicates the number of rabbits. Carrier cells, AdE3-*IAI.3B*-infected

A549 cells.

Table S6. Results of histopathological tests at 4 weeks after the final injections of carrier cells following chronic toxicity tests in rabbits

|  |
| --- |

Control A549 AdE3-*IAI.3B* Carrier cells (dose)

low moderate high

N 4 4 4 4 4 3

|  |
| --- |

Subcutaneous ----- + (1) ----- ----- ----- -----

hemorrhage

Lung edema ----- + (1) + (1) ----- ----- -----

Liver cirrhosis ----- ----- ----- ----- ----- -----

Liver inflammation ----- ----- ----- ----- ----- + (1)

Splenomegaly ----- ----- ----- + (3) + (2) + (2)

|  |
| --- |

Number in parentheses indicates the number of rabbits. Carrier cells, AdE3-*IAI.3B*-infected

A549 cells.

Table S7. Results of hematology tests following chronic toxicity tests in rabbits

|  |
| --- |

Test Date Control A549 AdE3-*IAI.3B* Carrier cells (dose)

low moderate high

|  |
| --- |

WBC Pre 9.7±2.5 9.4±3.3 9.1±2.3 8.4±2.4 8.4±2.4 8.4±2.4

(10^9^/L) Week 2 9.6±2.2 10.2±2.6 11.0±4.0 12.1±1.6* 12.6±1.9* 13.8±2.7*

Week 4 6.5±1.6 6.6±2.1 6.6±1.8 7.3±1.7 7.2±2.5 6.6±0.7

Week 8 4.9±1.2 5.1±0.3 6.1±1.7 6.0±2.3 6.7±1.4 6.1±1.2

RBC Pre 5.5±0.5 5.1±0.4 5.2±0.6 5.7±1.0 5.7±1.0 5.6±0.5

(10^12^/L)Week 2 5.0±0.5 5.0±0.9 5.0±0.5 4.9±0.6 4.7±0.6 4.3±0.6*

Week 4 5.5±0.3 5.2±1.0 5.7±0.6 5.4±0.5 5.6±0.5 5.0±0.4*

Week 8 5.7±0.3 5.9±0.3 6.2±0.3 6.6±0.8 6.1±0.7 6.1±0.5

HGB Pre 116±12 109±4 110±12 121±14 120±22 117±9

(g/L) Week 2 106±11 104±14 101±8 102±8 94±13* 88±11*

Week 4 116±8 104±20 113±0.6 107±8* 107±10* 99±7*

Week 8 122±5 119±14 129±6 132±12 117±8 124±13

HCT Pre 38.9±3.7 36,0±1.4* 37.3±3.0 40.0±5.1 39.3±5.1 38.8±2.5

(%) Week 2 35.9±4.0 35.0±5.2 35.0±2.6 35.4±2.4 33.3±4.2 32.7±4.1

Week 4 38.4±2.7 35.1±6.2 38.0±2.9 36.4±2.1 36.8±3.1 34.7±1.7*

Week 8 39.7±2.0 39.0±4.5 41,8±2.2 42.6±2.6 38.6±3.3 40.1±2.5

Neut Pre 3.9±1.8 4.8±2.4 3.3±1.4 3.0±1.4 3.5±1.4 3.9±2.5

(10^9^/L) Week 2 3.9±1.6 4.5±2.1 4.2±2.0 5.4±1.5* 6.1±1.5* 6.0±2.0*

Week 4 2.0±0.6 2.6±1.0 2.1±0.8 2.9±1.2 2.9±1.2 1.5±0.8

Week 8 1.6±0.6 1.9±0.3 1.9±1.4 2.4±0.7 2.6±1.3 1.7±0.3

PLT Pre 725±255 587±169 689±348 464±250* 515±291 750±340

(10^9^/L) Week 2 695±246 654±180 688±167 518±197 533±220 418±231*

Week 4 407±98 446±139 469±170 400±170 362±135 292±71*

Week 8 371±110 426±67 394±108 412±156 464±54 392±125

|  |
| --- |

WBC, white blood cell; RBC, red blood cell; HGB, hemoglobin; HCT, hematocrit; Neut, neutrophil; PLT, platelet; Carrier cells, AdE3-*IAI.3B*-infected A549 cells; *, *P* < 0.05.

Table S8. Results of hemostatic tests following chronic toxicity tests in rabbits

|  |
| --- |

Test Date Control A549 AdE3-*IAI.3B* Carrier cells (dose)

low moderate high

|  |
| --- |

PT Pre 10.1±0.6 9.9±0.5 9.9±0.3 10.4±1.0 10.2±0.7 9.8±0.5

(sec) Week 2 10.2±0.4 10.0±0.6 10.0±0.5 10.1±0.6 9.9±0.4 9.7±0.3

Week 4 10.4±0.4 10.1±0.2 10.3±0.3 10.0±0.4 10.2±0.6 10.2±0.2

Week 8 11.7±1.5 11.2±0.3 11.7±0.4 11.4±0.7 11.2±0.4 11.7±0.4

APTT Pre 24.3±5.3 23.2±5.0 22.6±4.8 29.6±15.0 25.8±8.0 21.8±5.3

(sec) Week 2 24.8±8.8 23.4±4.0 22.1±4.9 23.6±4.8 24.1±7.6 25.5±5.2

Week 4 25.2±8.8 23.6±6.8 23.4±7.6 21.9±3.8 21.7±5.4 19.9±4.9

Week 8 39.4±12.5 37.3±6.5 31.9±4.5 32.6±6.7 31.7±12.8 26.9±3.2

Fbg Pre 2.5±0.4 2.5±0.6 2.7±0.4 2.2±0.6 2.3±0.6 2.7±0.7

(g/L) Week 2 2.1±0.4 2.8±0.6* 2.4±0.4 3.4±0.6* 4.2±0.8* 4.8±0.7*

Week 4 2.2±0.3 3.1±0.6* 2.4±0.5 4.3±1.0* 4.9±3.0* 3.6±0.3*

Week 8 2.0±0.5 1.8±0.4 1.9±0.2 2.0±0.2 2.0±0.2 1.9±0.2

TT Pre 18.6±1.6 17.2±2.2 17.0±2.4 18.0±2.5 17.2±2.1 19.3±1.9

(sec) Week 2 17.8±2.8 18.1±2.4 17.4±1.8 18.8±2.7 19.5±2.4 20.1±2.2

Week 4 16.7±1.6 21.9±8.7* 17.2±2.8 19.9±2.5* 21.3±2.3* 20.3±1.4*

Week 8 21.2±4.9 17.9±4.0 18.4±0.7 20.9±0.9 18.8±1.7 19.5±0.7

|  |
| --- |

PT, prothrombin time; APTT, activated partial thromboplastin time; Fbg; fibrinogen; TT, thrombin time; Carrier cells, AdE3-*IAI.3B*-infected A549 cells;*, *P* < 0.05*.*

Table S9. Results of serum biochemical tests following chronic toxicity tests in rabbits

|  |
| --- |

Test Date Control A549 AdE3-*IAI.3B* Carrier cells (dose)

low moderate high

|  |
| --- |

ALT Pre 57±23 58±14 51±14 58±19 63±24 58±22

(U/L) Week 2 59±18 73±39 55±11 43±8* 53±6 52±21

Week 4 96±41 67±20 67±17 56±18* 55±12* 63±14*

Week 8 113±27 80±22 79±25 67±39 114±11 75±27

AST Pre 43±10 42±13 43±8 48±14 45±6 51±21

(U/L) Week 2 41±10 44±10 38±10 43±10 48±13 44±19

Week 4 64±30 43±19 46±17 50±17 41±9* 42±4

Week 8 66±16 53±7 40±5* 55±34 90±29 41±19

ALP Pre 97±52 89±31 77±26 112±21 95±45 92±34

(U/L) Week 2 75±38 62±21 68±24 51±16 46±19* 38±16*

Week 4 80±19 65±20 80±21 52±13* 51±17* 56±19*

Week 8 112±26 105±24 103±13 98±16 129±44 99±18

γ-GTP Pre 5.0±1.5 5.7±2.8 4.2±2.8 6.7±3.2 5.6±2.9 5.4±2.7

(U/L) Week 2 5.2±1.9 6.4±4.5 5.2±2.1 4.2±2.0 4.8±2.1 5.0±3.8

Week 4 7.5±2.9 5.9±5.3 5.8±1.7 3.4±2.0* 4.2±2.0* 4.4±2.9*

Week 8 8.0±1.4 8.0±5.2 8.5±2.4 7.0±1.4 8.5±1.0* 5.7±1.5

TP Pre 62.9±6.5 62.7±6.7 60.9±8.5 68.9±9.3 59.4±11.8 62.3±34

(g/L) Week 2 65.9±3.9 66.1±6.5 65.8±7.8 67.3±5.5 66.8±4.5 65.1±8.2

Week 4 67.4±4.0 72.2±5.9 69.0±5.7 75.3±4.7* 74.4±5.4* 84.5±5.4*

Week 8 65.3±7.2 67.6±1.7 65.0±2.3 68.3±3.7 68.3±3.4 69.8±2.4

ALB Pre 19.4±3.6 19.8±3.3 18.5±4.4 22.3±2.7 19.4±3.9 19.7±3.6

(g/L) Week 2 21.7±2.6 20.7±3.7 20.8±2.8 19.4±2.1* 18.6±1.6* 16.8±2.5*

Week 4 27.7±1.8 26.0±2.9 26.8±1.6 24.8±1.0* 23.3±1.9* 24.3±1.5*

Week 8 27.3±2.2 27.4±1.0 26.2±0.5 26.4±2.8 26.6±0.6 26.7±0.8

|  |
| --- |

ALT, alanine aminotransferase; AST, aspartate aminotransferase; ALP, alkaline phosphatase;

γ-GTP, gamma-glutamyl transpeptidase; TP, total protein; ALB, albumin; Carrier cells, AdE3-*IAI.3B*-infected A549 cells; *, *P* < 0.05*.*

Table S10. Results of serum biochemical tests following chronic toxicity tests in rabbits

|  |
| --- |

Test Date Control A549 AdE3-*IAI.3B* Carrier cells (dose)

low moderate high

|  |
| --- |

TCHO Pre 1.0±0.3 1.2±0.4 1.5±0.6* 1.3±0.4 1.3±0.4 1.2±0.5

(mmol/L)Week 2 1.3±0.8 1.5±0.5 1.3±0.3 1.4±0.5 1.8±1.2 2.7±1.6*

Week 4 1.1±0.2 1.3±0.4 1.4±0.3* 1.8±0.6* 2.3±1.3 2.4±1.1

Week 8 1.4±0.4 1.7±0.1 2.3±0.6* 1.8±0.3 2.2±0.8 2.1±0.6*

TG Pre 1.3±0.5 1.2±0.7 2.1±1.3 1.2±0.7 1.5±1.3 1.5±1.3

(mmol/L)Week 2 1.7±0.6 2.0±0.7 1.9±0.7 1.9±0.5 1.9±0.7 3.4±1.9*

Week 4 1.2±0.5 1.3±0.4 1.2±0.3 1.7±0.6 1.6±0.5 2.2±0.7*

Week 8 1.5±0.3 1.0±0.4 1.4±0.6 1.1±0.4 1.3±0.1 1.4±0.4

BUN Pre 8.3±1.2 8.3±2.0 7.9±1.7 7.2±2.4 8.8±1.8 8.4±2.1

(mmol/L)Week 2 6.2±2.4 7.1±1.2 6.0±1.3 5.8±1.1 6.2±1.1 7.1±4.1

Week 4 9.6±3.0 9.2±2.0 8.9±2.7 8.8±1.6 8.0±1.8 8.8±3.1

Week 8 7.1±1.0 6.3±0.2 6.5±0.6 6.9±0.4 6.7±0.8 6.9±1.2

Creat Pre 107±11 102±11 106±18 113±16 111±18 109±15

(μmol/L) Week 2 93±10 89±12 98±10 95±18 91±9 70±27*

Week 4 125±18 110±9 122±15 113±16 113±14 109±16

Week 8 124±11 131±15 137±6 138±12 133±6 134±18

CK Pre 2275±1037 2432±1246 2381±865 2334±899 2803±1347 2390±1223

(U/L) Week 2 2701±1512 2826±1321 2100±816 3450±903 3355±1221 3262±1638

Week 4 1582±508 1260±534 1574±700 2331±882* 1985±858* 1639±1323

Week 8 922±154 1191±163 1357±511 1091±536 1591±499 1019±150

GLU Pre 5.8±1.1 6.3±1.3 5.2±1.6 5.8±1.5 5.9±1.2 6.2±1.7

(μmol/L) Week 2 5.0±1.0 5.4±1.2 5.2±0.7 5.1±1.1 5.3±1.1 5.7±1.3

Week 4 5.9±0.9 5.9±1.0 5.7±1.5 7.3±2.5 6.1±1.9 6.3±1.3

Week 8 6.2±0.7 5.9±0.5 6.0±0.2 5.7±0.8 5.9±0.9 6.2±1.2

|  |
| --- |

TCHO, total cholesterol; TG, triglyceride; BUN, blood urea nitrogen; Creat, creatinine; CK,

creatine kinase; GLU, glucose; Carrier cells, AdE3-*IAI.3B*-infected A549 cells; *, *P* < 0.05.
